# beachmat: A Bioconductor C++ API for accessing high-throughput biological data from a variety of R matrix types

**DOI:** 10.1371/journal.pcbi.1006135

**Published:** 2018-05-03

**Authors:** Aaron T. L. Lun, Hervé Pagès, Mike L. Smith

**Affiliations:** 1 Cancer Research UK Cambridge Institute, University of Cambridge, Li Ka Shing Centre, Cambridge, United Kingdom; 2 Fred Hutchinson Cancer Research Center, Seattle, Washington, United States of America; 3 European Molecular Biology Laboratory (EMBL), Genome Biology Unit, Heidelberg, Germany; Johns Hopkins University, UNITED STATES

## Abstract

Biological experiments involving genomics or other high-throughput assays typically yield a data matrix that can be explored and analyzed using the R programming language with packages from the Bioconductor project. Improvements in the throughput of these assays have resulted in an explosion of data even from routine experiments, which poses a challenge to the existing computational infrastructure for statistical data analysis. For example, single-cell RNA sequencing (scRNA-seq) experiments frequently generate large matrices containing expression values for each gene in each cell, requiring sparse or file-backed representations for memory-efficient manipulation in R. These alternative representations are not easily compatible with high-performance C++ code used for computationally intensive tasks in existing R/Bioconductor packages. Here, we describe a C++ interface named *beachmat*, which enables agnostic data access from various matrix representations. This allows package developers to write efficient C++ code that is interoperable with dense, sparse and file-backed matrices, amongst others. We evaluated the performance of *beachmat* for accessing data from each matrix representation using both simulated and real scRNA-seq data, and defined a clear memory/speed trade-off to motivate the choice of an appropriate representation. We also demonstrate how beachmat can be incorporated into the code of other packages to drive analyses of a very large scRNA-seq data set.

This is a *PLOS Computational Biology* Software paper.

## Introduction

The combination of the statistical programming language R [1] and the open-source Bioconductor project [2] represents a key platform for exploring and analyzing high-throughput biological data. R provides efficient, rigorously tested, open-source implementations of many statistical and numerical procedures. Its interactive nature lends itself to data exploration and research, while its programming features allow assembly of complex analyses. It is also extensible through the installation of optional “packages”, often contributed by the research community, which contain bespoke methods for specific scientific problems. In particular, the Bioconductor project [3] provides over a thousand packages for analyzing biological data in fields such as genomics, transcriptomics and proteomics. Packages are mostly written in R but can also include native code (in C/C++ or Fortran) for computationally intensive tasks. Use of native C++ code is facilitated by the *Rcpp* package [4], which simplifies the integration of package code with the R application programming interface (API).

A matrix of measurements is a common starting point in many analysis workflows for high-throughput biological data. A typical example is the expression matrix in transcriptomics data, where each row represents a gene, each column represents a sample and each entry represents the quantified expression (e.g., number of mapped reads, transcripts-per-million) for a gene in a sample. By default, this is represented in R as an “ordinary” matrix, where each entry is explicitly stored in random access memory (RAM) in a dense contiguous array. Alternatively, it can be represented as a sparse matrix using classes from the *Matrix* package [5], which saves memory by only storing non-zero entries. Another option is to use file-backed representations such as those in the *bigmemory* [6] or *HDF5Array* packages, where the data are stored in a file and parts of it are loaded into RAM upon request. For each representation, methods are provided in R for common operations such as subsetting, transposition and arithmetic, such that any downstream code for data processing can be agnostic to the exact representation of the matrix. This simplifies software development and improves interoperability.

The major benefit of alternative representations is that they allow efficient handling of large matrices in R without using large amounts of RAM to construct a dense array. This is particularly important for the effective analysis of biological data sets due to the increasing throughput of the experimental protocols. It is well known that the output of DNA sequencing machines has risen consistently over the past decade [7], which is compounded by the increasing complexity of the assays. For example, droplet-based protocols in single-cell transcriptomics [8–10] generate transcriptome-wide expression profiles for each of thousands or even millions of cells. Similar issues are encountered outside of transcriptomics, with single-cell ATAC-seq [11] and bisulfite sequencing [12] yielding genome-wide data (from individual genomic regions or base positions, respectively) for each cell. Analyses of these large data sets often involve an extended period of interactive data exploration where the matrix might be transformed, subsetted or rearranged. Given R’s copy-on-write semantics, matrix representations that achieve efficient memory usage throughout the course of an analysis are highly desirable.

Unfortunately, the use of alternative matrix representations is less straightforward for compiled code written in statically typed languages like C++. Existing interfaces for reading R matrices in C/C++ require the details of the matrix representation to be specified during compilation. This makes it difficult to write a single, general piece of code that can be applied to many different representations. Writing multiple versions of a function for different representations is difficult and unsustainable when more representations become available. The other option is to perform all processing in R to exploit the availability of common methods. However, this is an unappealing strategy for high-performance code. R is often slower than C++ by at least an order of magnitude for arbitrary programming tasks that cannot be “vectorized”, i.e., made to operate on all elements of a vector at once. This includes common procedures in biological data analysis such as looping across all samples or genes and performing arbitrarily complex operations on the sample- or gene-specific observations. The use of R alone would increase the computational time required to perform analyses, which is inconvenient for interactive analyses and unacceptable for large simulation studies. It would clearly be preferable to implement critical functions in native code wherever possible.

Here, we describe a C++ API named *beachmat* (using Bioconductor to handle Each Matrix Type), which enables C++ code to access R matrix data in a manner that is agnostic to the exact matrix representation. This allows package developers to implement computationally intensive algorithms in C++ that can be immediately applied to a wide range of R matrix classes including ordinary matrices, sparse matrices from the *Matrix* package, and HDF5-backed matrices from the *HDF5Array* package. Using simulated and real transcriptomics data, we assess the performance of *beachmat* for data access from each matrix representation. We show that each representation has specific strengths and weaknesses, with a clear memory-speed trade-off that motivates the use of alternative representations in different settings. We also demonstrate how *beachmat* can be used by other Bioconductor packages to enable the analysis of a very large single-cell RNA sequencing (scRNA-seq) data set. By operating synergistically with existing Bioconductor infrastructure, *beachmat* extends R’s capabilities for analyzing high-throughput biological data stored in large matrices.

## Design and implementation

### Overview

**Fig 1:**
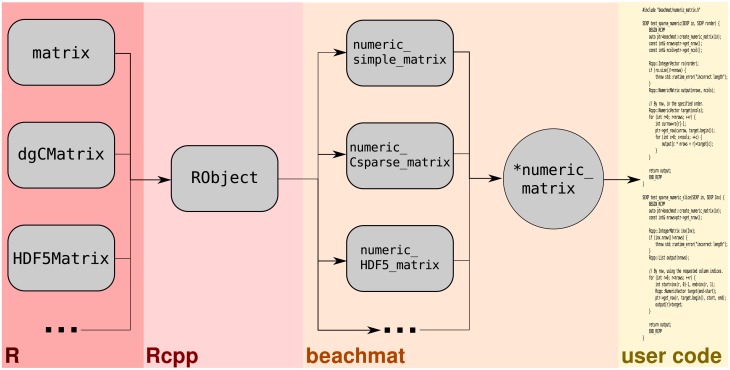
Schematic of the *beachmat* workflow. Various matrix representations at the R level are passed as RObject instances to a C++ function. *beachmat* identifies the specific representation, constructs an instance of the appropriate C++ derived class, and returns a pointer to the base class. (In this case, a numeric_matrix pointer is returned for input matrices holding double-precision data.) This pointer can then be used in user-level code in a manner that is agnostic to the details of the original representation.

The *beachmat* API uses C++ classes to provide a common interface for data access from R matrix representations. For all representations of a given data type (e.g., integer, double-precision, character strings), we define a base class with common methods for data access. Each specific representation is associated with a derived class that provides customized implementations of the access methods. The intention is for a user to pass in an R matrix of any type, in the form of an RObject instance from the *Rcpp* API (Fig 1). *beachmat* then constructs an instance of the appropriate derived class, returning a pointer to the base class. This pointer is the same regardless of the representation and can be used in downstream C++ code to achieve run-time polymorphism.

When access to a specific row or column (or a part thereof) is requested, the *beachmat* API will use representation-specific methods to fill a *Rcpp*-style Vector object with corresponding data values from the matrix. This copy-on-access strategy ensures that the API behaves consistently across different matrix representations, and provides flexibility for downstream applications by allowing in-place modifications to vector elements and guaranteeing contiguous data storage. It is also possible to avoid copying altogether when performing read-only data access from columns of ordinary or sparse matrices, improving efficiency in certain situations. Finally, a request for a specific entry of the matrix will directly return the corresponding data value.

While the *beachmat* API is agnostic to the matrix representation, it still needs to know the type of data that are stored in the matrix. We use C++ templating to recycle the code to define specific classes for common data types, i.e., logical, integer, double-precision floating point or character strings. The same methods are available for all classes of each data type, improving their ease of use for developers.

### Supported matrix representations

The simplest matrix representation is the ordinary R matrix, where data are stored in RAM as a contiguous dense array in column-major format. This is most commonly constructed with the base matrix function, though the dgeMatrix class from the *Matrix* package implements an equivalent representation. Both of these classes are supported by *beachmat*. Using simulated data (Section 1 of S1 Text), we measured the speed of row- or column-level data access from ordinary matrices with *beachmat*. Our results indicate that *beachmat* provides comparable performance to a reference *Rcpp* implementation (Section 2 of S1 Text, S1 Fig).

**Fig 2:**
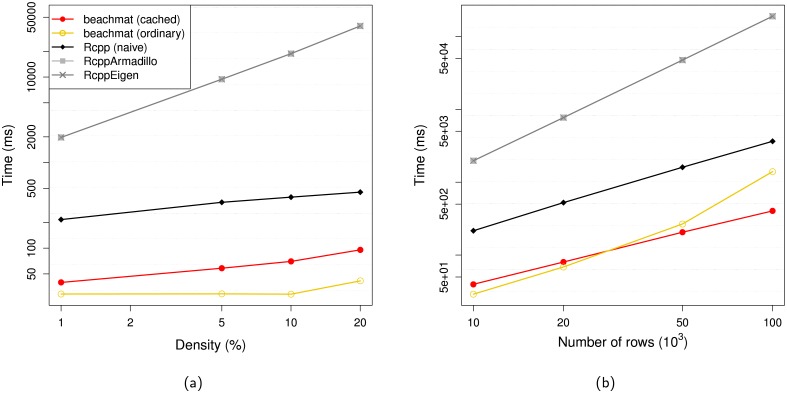
Timing of access to consecutive rows of simulated CSC matrices using the caching method in *beachmat*, a naive binary search written in *Rcpp* or through the *RcppArmadillo* and *RcppEigen* APIs. For reference, the access time for an equivalent ordinary matrix is also shown. (a) Access times with respect to the density of non-zero entries, for a matrix with 10000 rows and 1000 columns. (b) Access times with respect to the number of rows, for a matrix with 1000 columns and 1% non-zero entries. Each value shown above represents the time required to access all rows of the matrix, averaged across 10 iterations. Horizontal dotted lines represent 2-fold increases in time.

*beachmat* also supports data access from sparse matrices in the compressed sparse column-orientated (CSC) format (Section 3.1 of S1 Text, S2 Fig), implemented in the dgCMatrix class from *Matrix*. This representation only stores non-zero values in RAM, which is highly memory-efficient for assays that yield many zeroes, e.g., scRNA-seq. When the density of non-zero entries is low, column access from CSC matrices can also be faster than the equivalent ordinary matrix (Section 3.2 of S1 Text, S3 Fig). However, row access from CSC matrices is more complex, as obtaining elements from an arbitrary row of the matrix involves a binary search for each column (Section 3.3 of S1 Text). We use a novel caching strategy to improve the efficiency of accessing data from consecutive rows by avoiding unnecessary binary searches (Fig 2). This provides a 4-fold increase in speed compared to a naive approach, as well as faster row-level access than the *RcppArmadillo* and *RcppEigen* APIs [13, 14]. Our caching strategy also improves performance for non-consecutive row access (S4 Fig).

**Fig 3:**
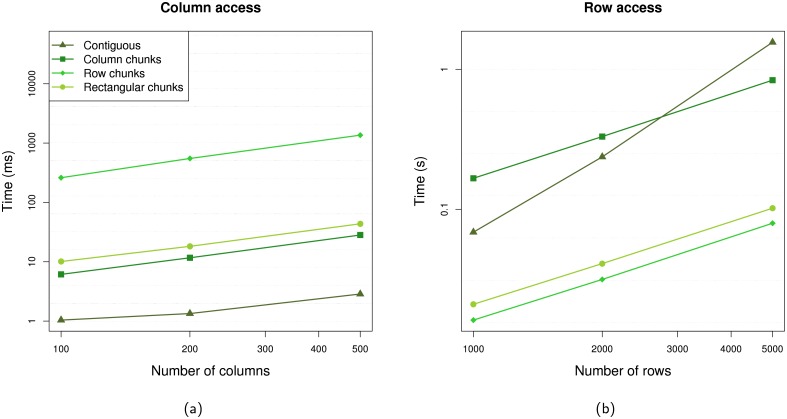
Timing of *beachmat*-based access to consecutive columns or rows of a simulated HDF5Matrix object constructed with different HDF5 file layouts, i.e., contiguous, row- or column-chunking, or 40 × 40 rectangular chunks. (a) Column access times with respect to the number of columns, for a matrix with 1000 rows. (b) Row access times with respect to the number of rows, for a matrix with 100 columns. Each value shown above represents the time required to access the entirety of the matrix, averaged across 10 iterations. Horizontal dotted lines represent 2-fold increases in time.

Another representation supported by *beachmat* is a file-backed matrix based on the hierarchical data format (HDF5) [15], implemented in the HDF5Matrix class from the *HDF5Array* package (Section 4 of S1 Text). This stores the entire data set in a HDF5 file, only retrieving subsets into RAM upon request. As a result, it is very memory-efficient but much slower than the other representations (S5 Fig). A key determinant of access speed is the layout of the HDF5 file, where data can be split into “chunks” for easier retrieval. Once read, chunks can be cached in memory to avoid redundant retrieval of data from the same chunk in adjacent rows or columns. To improve performance, *beachmat* automatically tunes the parameters of the HDF5 chunk cache to optimize data access from consecutive rows or columns (S2 Text). This allows *beachmat* to use the same rectangular chunking layout for row and column access (Fig 3) with performance comparable to pure row- and column-chunking layouts. We also provide functions to choose suitable chunk dimensions during file creation, when consecutive row or column access is expected downstream; as well as functions to convert to layouts that are well-suited for random row or column access (S6 and S7 Figs, S3 Text).

Other representations are also supported, including packed symmetric matrices and matrices based on run-length encodings (see Section 5 of S1 Text for details). As a general rule, matrix representations that occupy more RAM provide faster data access, as data do not need to be unpacked or retrieved from file. The exception is that of sparse matrices with few non-zero entries, where the ability to ignore zeroes can dramatically improve performance for certain algorithms. Indeed, for a more complex operation like matrix multiplication, the use of sparse matrices is much faster than ordinary or HDF5-backed matrices (Section 6 of S1 Text, S8 Fig), though obviously this depends on the density of non-zero entries.

### Generating matrix output from C++

In addition to accessing data in existing matrices, the *beachmat* API allows C++ code to store data in various representations for output to R. For integer, logical, double-precision and character data, ordinary and HDF5-backed matrices can be constructed that are indistinguishable from those generated in R. Logical and double-precision data can also be stored in sparse format, where only true or non-zero values are retained in lgCMatrix or dgCMatrix instances, respectively. (The *Matrix* package does not support sparse integer or character matrices, so these are not considered.) The output representation can either be explicitly specified in the code, or it can be automatically chosen to match some input representation. To illustrate, consider a C++ function that accepts a matrix as input and returns a matrix of similar dimensions. If the input is an ordinary matrix, one might assume that there is enough RAM to also store the output as an ordinary matrix; whereas if the input is a HDF5Matrix, one could presume that the output would be similarly large, thus requiring a HDF5Matrix representation for the results. This means that results of processing in C++ can be returned in the most suitable representation for manipulation in R.

### Alternative strategies for matrix manipulation

An alternative to using *beachmat* is to write C++ code for ordinary matrices and apply it to submatrices (or “blocks”) of a given input matrix. Each block is coerced to an ordinary matrix before it is supplied to the C++ code. After looping across all blocks, the block-wise results are combined to obtain the final result for the entire matrix. This block processing strategy allows the application of C++ code while controlling RAM usage by storing only one block as an ordinary matrix at any given time. However, it requires coordination between R and C++ to keep track of the block that is being processed, to monitor intermediate variables that persist between blocks, and to combine the results in an appropriate manner. The need to ensure that R and C++ are interacting correctly at multiple points imposes a significant burden on the developer. Computational efficiency is also reduced by looping in R, multiple matrix coercions and repeated C++ function calls. The *beachmat* API provides a natural solution by moving the entire procedure into C++, simplifying development and maintenance.

Unlike the *RcppArmadillo* and *RcppEigen* APIs, *beachmat* does not provide any direct support for matrix operations such as multiplication or factorization. Rather, *beachmat* was developed for applications that treat an input matrix as a collection of (otherwise separate) column- or row-wise vectors. This is a common use case for data matrices generated by high-throughput biological experiments, where a function may need to loop over rows or columns to perform calculations for each assayed feature or sample, respectively. Indeed, most Bioconductor packages that use C++ (309 packages in release 3.6, based on the requirement for *Rcpp*) do not use *RcppEigen* or *RcppArmadillo* (required by 8 and 74 packages, respectively). This suggests that the lack of support for matrix operations in *beachmat* will not be a major hindrance to its utility in the Bioconductor development framework. In fact, a representation-agnostic API may not be practical for matrix operations that are not scalable for very large file-backed matrices. Switching to faster approximate approaches is a decision for the developer/user, not a low-level API like *beachmat*.

## Results

### Use cases in single-cell RNA sequencing

Recent advances in scRNA-seq technologies have led to an explosion in the quantity of data that can be generated in routine experiments. Droplet-based methods such as Drop-Seq [8], inDrop [9] and GemCode [10] allow transcriptome-wide expression profiles involving 10,000-40,000 genes to be captured in each of thousands to millions of cells. Careful computational analysis is critical to extract meaningful biology from these data, but their sheer volume strains existing pipelines and methods designed for single-cell data processing. The data analysis challenge is compounded by the presence of large-scale projects such as the Human Cell Atlas [16], which aims to use single-cell ‘omics to profile every cell type in the human body. The increasing size of these data sets motivates the use of alternative matrix representations to store the data as well as the efficient implementation of analysis methods in native code. This presents itself as a highly relevant use case for *beachmat*, which we will explore in the following sections.

### Access times for a small brain data set

We evaluated the performance of *beachmat* with the different matrix representations on real scRNA-seq data from a study of the mouse brain by Zeisel *et al*. [17] (Section 1 of S4 Text). This data set contains integer expression values for 19972 genes in each of 3005 brain cells, derived from different regions of the brain and consisting of a diverse range of cell types. Only 18% of expression values in the matrix are non-zero, reflecting the low capture efficiencies in single-cell transcriptomics experiments [18]. The Zeisel *et al*. data set is not particularly large, especially in the context of droplet-based experiments that routinely generate transcriptome-wide data for tens of thousands of cells. However, for our purposes, the smaller number of cells in this data set is desirable as it means that each of the matrix representations—including those that are stored wholly in RAM—can be easily evaluated and compared.

**Fig 4:**
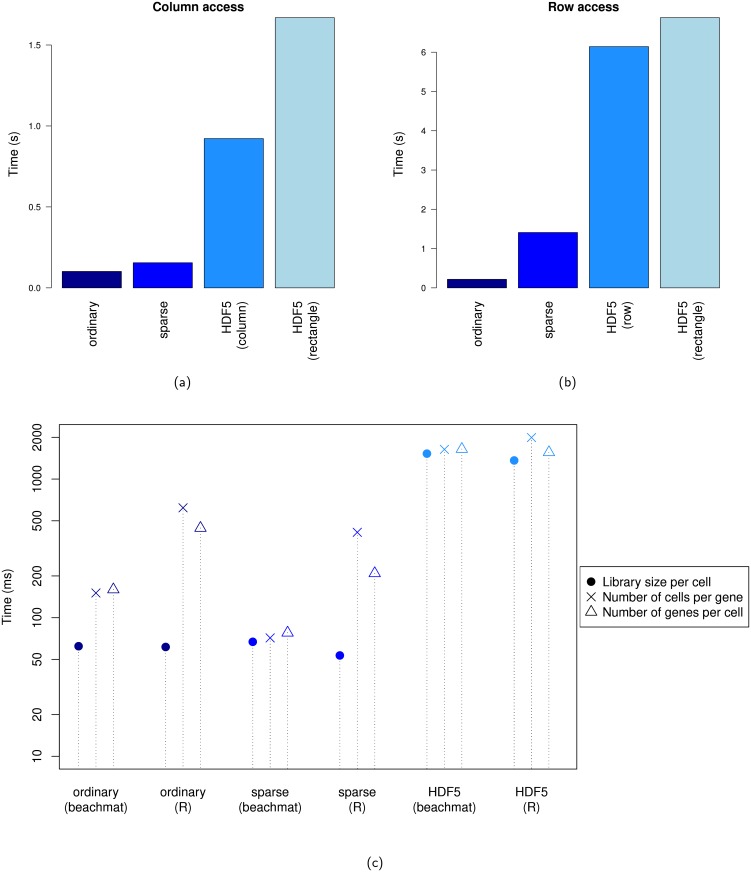
Timing for data access and calculation of common metrics from various matrix representations of the mouse brain data set from Zeisel *et al*. [17]. (a) Time required to calculate the column sums for each representation by accessing data from each column. For HDF5Matrix, a column-chunked layout and a rectangular 200 × 200 layout were tested. (b) Time required to calculate the row sums for each representation by accessing data from each row. For HDF5Matrix, a row-chunked layout and a rectangular 200 × 200 layout were tested. (c) Time required to compute the library size per cell, the number of cells expressing each gene or the number of genes expressed in each cell, using C++ functions written with *beachmat* or the relevant built-in methods in R. Each value is an average of 10 repeated timings; standard errors were negligible and are not shown.

We converted the Zeisel *et al*. data to the various matrix representations and measured the access speed for the row- or column-level data with *beachmat*. We observed similar results to those obtained with simulated data, recapitulating the different trade-offs between access speed and RAM usage across representations. Specifically, row and column accesses from an ordinary matrix were fastest, followed by accesses from a sparse matrix (Fig 4a and 4b). HDF5-backed matrices provided slowest access but also the smallest memory footprint (2 KB, compared to 480 MB for ordinary matrices and 130 MB for sparse matrices). The size of the HDF5 file was relatively small, requiring only 16-20 MB of space for each HDF5Matrix instance. We also recorded the time required to compute some gene- or cell-specific metrics (Fig 4c, Section 1 of S4 Text) commonly used in scRNA-seq data analysis. Our custom C++ functions based on *beachmat* were at least comparable to the built-in R functions, and were faster in some cases by avoiding a type conversion to a logical matrix.

An appropriate choice of matrix representation depends on the context in which it is used. We could use purely in-RAM representations for optimal access speed, but this may not be practical for very large data sets. Even high-performance computing systems have their limits, especially when multiple copies of the matrix are generated throughout the course of an analysis. Sparse representations also become ineffective if sparsity-destroying operations (e.g., mean-centering, batch correction) are applied. In such cases, it may be preferable to sacrifice speed for reduced memory consumption by using file-backed representations such as HDF5Matrix. By incorporating *beachmat* into the C++ code, an R package can dynamically accept different matrix types appropriate for the size of the data set and computing environment.

### Analysis of the large 10X data set

**Fig 5:**
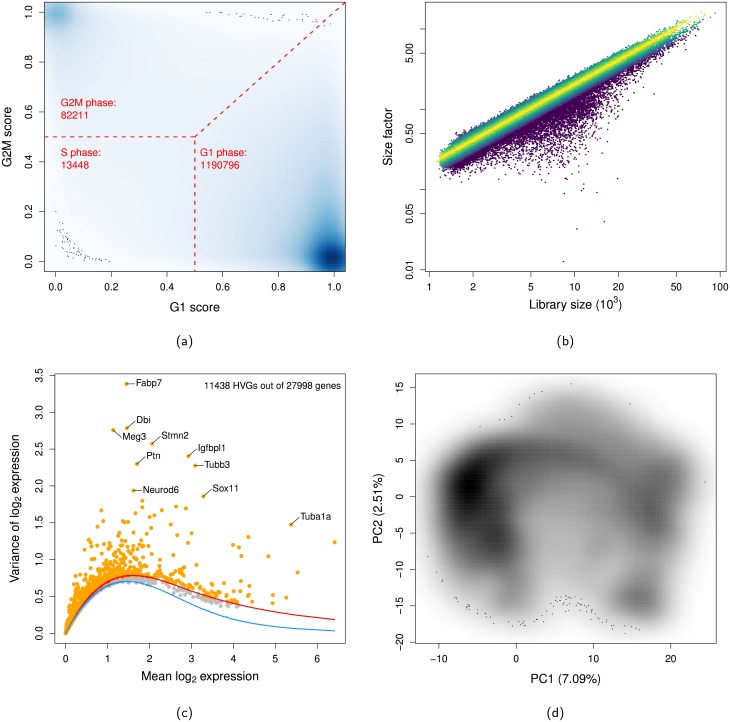
Analysis of the 10X 1.3 million brain cell data set. (a) Cell cycle phase assignment, based on the G1 and G2M scores reported by cyclone. The intensity of colour is proportional to the density of cells at each plot location. Dashed lines indicate the score boundaries corresponding to each phase, and the number of assigned cells is also shown for each phase. (b) Size factor for each cell from the deconvolution method, plotted against the library size. Cells are coloured according to the deviation from the median log-ratio of the size factor to the library size across all cells. (c) Variance of the normalized log-expression values for each gene, plotted against the mean log-expression. The red line represents the mean-dependent trend fitted to all genes, while the blue line represents the mean-variance trend corresponding to Poisson noise. Orange points represent HVGs with variances above the red line, with the top 10 genes highlighted. (d) PCA plot generated from the HVG expression profiles of all cells. The variance explained by each of the first two principal components is shown in brackets.

To demonstrate the utility of *beachmat* for faciliting analyses of large data sets, we converted several functions in the *scater* [19] and *scran* packges [20] to use *beachmat* in their C++ code. We applied these functions to the 1.3 million brain cell data set from 10X Genomics (Section 2 of S4 Text). First, we called cell cycle phase with the cyclone method [21], which required access to each column (i.e., single cell) of the count matrix via *beachmat*. Most cells were identified as being in G1 phase (Fig 5a), consistent with the presence of differentiated neurons that are not actively cycling. Next, we applied the deconvolution method [22] to normalize for cell-specific biases, again using column-level data access. This yielded a size factor for each cell, which was generally well-correlated with the library size (Fig 5b). However, a few cells have size factors that are much smaller than expected based on their library sizes, and there is a modest amount of scatter around the size factor-library size trend. This is consistent with composition effects [23] caused by differential gene expression between cell subpopulations.

We detected highly variable genes (HVGs) based on the variance of the log-normalized expression values for each gene [20]. We blocked on the sequencing library of origin for each cell to regress out technical factors of variation unrelated to biological heterogeneity. This was done on a gene-by-gene basis, and thus required row-level access to the log-expression matrix via *beachmat*. We identified a number of HVGs (Fig 5c), including genes involved in neuronal differentiation and function such as *Neurod6* [24] and *Sox11* [25]. Finally, we performed dimensionality reduction on the HVG expression profiles for all cells using randomized principal components analysis (PCA) [26]. Visualization of the first two principal components (PCs) showed clear substructure in the cell population (Fig 5d), reflecting the diversity of cell types in the mouse brain. Indeed, once PCA has been performed, the first 10-100 PCs for each cell can be used as a summary of its expression profile. This can be stored as an ordinary matrix and supplied directly to other R functions for clustering [27, 28] or trajectory reconstruction [29]. At this point, memory usage ceases to be an issue and we only need to choose algorithms that are scalable with respect to the number of cells.

While a full characterisation of this data set is outside the scope of this article, it is clear that we can proceed through many parts of the scRNA-seq analysis pipeline using *beachmat*-driven C++ functions. By taking advantage of the file-backed HDF5Matrix, this analysis can be conducted in reasonable time on a desktop with modest specifications (see Section 3 of S4 Text). These features allow us to obtain biological insights that previously would have been inaccessible from R. We also note that the incorporation of the *beachmat* API only required small modifications to existing C++ code for scRNA-seq data analysis. This indicates that many established methods in the R/Bioconductor ecosystem can be easily and immediately extended to work with large data sets, enabling the statistically rigorous analysis of large single-cell data.

## Availability and future directions

The *beachmat* package contains the C++ API and is available as part of version 3.6 of the Bioconductor project (https://bioconductor.org/packages/beachmat). It is straightforward to integrate *beachmat* into existing R packages, enabling arbitrary C++ code to accept many different matrix inputs without any further effort on the part of the developer. Our modifications to the *scran* and *scater* packages have enabled the analysis of a very large scRNA-seq data set in low-memory environments using file-backed representations, without significantly compromising speed for smaller data sets that can be held fully in memory. These modifications are now implemented in the latest versions of *scran* and *scater*, both of which can also be installed from Bioconductor.

The popularity of the R programming language stems, in part, from the ease with which it can be extended. Packages can be easily developed by the research community to implement cutting-edge algorithms for new data types. The increasing number of packages designed to analyze single-cell data (43 on Bioconductor at time of writing) provides a case in point. We anticipate that *beachmat* will be useful to developers of novel computationally intensive bioinformatics methods that need to access data from different matrices. While we have focused on scRNA-seq in this paper, analyses of other large matrices (e.g., genome-wide contact matrices in Hi-C data [30]) may also benefit from *beachmat*-driven code. We will also continue to develop *beachmat* to support data access from new formats such as Loom for scRNA-seq data (http://loompy.org/).

We note that, for large single-cell data, the utility of *beachmat* for R package development ultimately depends on the scalability of the underlying algorithms for processing millions or even billions of cells. Most existing methods for scRNA-seq data analysis are designed to handle thousands of cells at best. Fortunately, we can make use of the existing expertise in the Bioconductor community to improve scalability. Highly multiplexed flow and mass cytometry experiments routinely generate low-dimensional data for millions of cells, and many Bioconductor packages are already available to analyze and interpret these data [31–33]. A low-rank representation of scRNA-seq data (e.g., after PCA) is similar to cytometric data in size and structure, suggesting that algorithms used in the latter can also be applied to the former. This presents an interesting avenue for future development of software based on *beachmat*.

All R/C++ code used to perform the simulations and real data analyses are also available on Github (https://github.com/LTLA/MatrixEval2017).

## Supporting information

S1 Text Performance of *beachmat* on different matrices. (PDF)

S2 Text Optimizing HDF5 chunk cache parameters. (PDF)

S3 Text Dealing with random access from a HDF5Matrix. (PDF)

S4 Text Methods for real data processing. (PDF)

S1 Fig Time required to access consecutive columns or rows of simulated ordinary matrices using the *beachmat* or *Rcpp* APIs. (a) Column access time with respect to the number of columns, for a matrix with 10000 rows. Times are shown for *beachmat* with and without copying of matrix data. (b) Row access time with respect to the number of rows, for a matrix with 1000 columns. Each timing represents the average of 10 simulations, and involves accessing the entirety of the matrix. Intervals between the horizontal dotted lines represent 2-fold increases in time.
(PDF)

S2 Fig A schematic of the column-sparse compressed matrix format, as implemented by the dgCMatrix class in the *Matrix* package. The grey box represents a sparse matrix with zero entries indicated by the dots. The x vector stores all non-zero values, ordered in column-major format. The index of each element in x is shown in red. The i vector stores the row indices (blue) corresponding to the ordered non-zero values. The p vector stores the element index of the first non-zero value in each column (brown). The last element of p is always the total number of non-zero entries.
(PDF)

S3 Fig Time required to access consecutive columns of simulated CSC matrices using *beachmat* or the *RcppArmadillo* and *RcppEigen* APIs, compared to an equivalent ordinary matrix with *beachmat*. Timings were also recorded for a copy-free column access method for CSC matrices in *beachmat*. (a) Access times with respect to the density of non-zero entries as a percentage of all entries, for a matrix with 10000 rows and 1000 columns. (b) Access times with respect to the number of columns, for a matrix with 10000 rows and 1% density. Each timing represents the average of 10 simulations, and involves accessing all columns in the matrix. Horizontal dotted lines represent 2-fold increases in time.
(PDF)

S4 Fig Time required to access non-consecutive rows of simulated CSC matrices using the caching method in *beachmat* or a naive binary search implemented in *Rcpp*, compared to an equivalent ordinary matrix with *beachmat*. (a) Access times for ordered but non-consecutive rows, with respect to the number of rows in a matrix with 10000 rows and 1000 columns at 1% density. This involved accessing every 5^th^ row and returning to the first unaccessed row, i.e., {1, 6, …, 9996, 2, 7, …}. (b) Access times for random rows, with respect to the number of rows in the matrix described previously. Each timing represents the average of 10 simulations, and involves accessing all rows in the matrix exactly once. Horizontal dotted lines represent 2-fold increases in time.
(PDF)

S5 Fig Time required for *beachmat* to access consecutive rows or columns of a simulated HDF5-backed matrix using column/row-chunking or rectangular 100 × 100 chunks, compared to an equivalent ordinary matrix. (a) Column access time with respect to the number of columns, for a dense matrix with 10000 rows. (b) Row access time with respect to the number of rows, for a dense matrix with 1000 columns. Each timing represents the average of 10 simulations, and involves accessing the entirety of the matrix. Horizontal dotted lines represent 2-fold increases in time.
(PDF)

S6 Fig Time required to use *beachmat* to access random rows or columns of a simulated HDF5-backed matrix constructed with different HDF5 file layouts, i.e., contiguous, row- or column-chunking, or 40 × 40 rectangular chunks. (a) Random column access times with respect to the number of columns, for a dense matrix with 1000 rows. (b) Random row access times with respect to the number of rows, for a dense matrix with 100 columns. Each row or column in the matrix was accessed exactly once in random order. Horizontal dotted lines represent 2-fold increases in time.
(PDF)

S7 Fig Time required to convert from a column-based chunk layout to a row-based chunk layout, or vice versa, in HDF5-backed matrices. Each chunk contained 5000 values along a single row or column (or set to the corresponding dimension of the matrix, if it was smaller than 5000). Conversion times were recorded with respect to increasing number of (a) columns for a dense matrix with 10000 rows, or (b) rows for a dense matrix with 1000 columns. All values represent the mean of 10 simulation iterations. Horizontal dotted lines represent 2-fold increases in time.
(PDF)

S8 Fig Time required to perform matrix multiplication between square ordinary matrices, between sparse matrices or between a HDF5-backed matrix and an ordinary matrix, as a function of the order of the matrix. Matrix multiplication was performed using a simple algorithm implemented in C++ with *beachmat*, or the representation-specific %*% operators in R. For sparse matrix multiplication, timings are also provided for an alternative algorithm implemented in *beachmat* that better exploits sparsity (II). Timings for the multiplication of two HDF5-backed matrices are shown for *beachmat* only, as the equivalent operation is not yet supported by *DelayedArray*.
(PDF)

S1 Code R and C++ code used for all simulations, timings and analyses. (ZIP)
