# Supplementary material for: beachmat: A Bioconductor C++ API for accessing high-throughput biological data from a variety of R matrix types
Source: PLoS Comput Biol. 2018 May 3;14(5):e1006135. doi: 10.1371/journal.pcbi.1006135 (PMC5953501; doi:10.1371/journal.pcbi.1006135)
Supplement: S1 Text — (PDF) [file pcbi.1006135.s001.pdf]

S1 Text: performance of *beachmat* on different matrices

Aaron T. L. Lun, Hervé Pagès, Mike L. Smith

April 19, 2018

1 Access timings with simulated data

Double-precision ordinary matrices of specified dimension were filled with values sampled from a standard normal distribution. All ordinary matrices were constructed using the `matrix` function. Double-precision CSC matrices of a specified dimension and density were generated as `dgCMatrix` instances, using the `rsparsematrix` function from the *Matrix* package. HDF5-backed matrices were generated using the `writeHDF5Array` function from the *HDF5Array* package on the ordinary matrices with explicitly specified chunk sizes. Conversion of each matrix object into other representations, if necessary, was performed prior to timing access with the C++ APIs. (This only refers to representations at the R level – any conversions in C++ were considered to be part of the API and timed accordingly.)

To time row and column access with *beachmat* and other APIs, we wrote C++ functions (one per API) that compute the sum of values in each row or column, respectively. This approach ensures that each entry of the row/column is visited in order to use its value, avoiding trivially fast approaches where a pointer or iterator is returned to the start of the column/row. Summation is also simple enough that the access time of the API will still constitute a major part of the overall time spent by the function. We stress that, in each run of the function, we are measuring the time required to access each data element of the entire matrix, not just from a single row or column of the matrix.

Timings were performed in R using the `system.time` function on a call to each C++ function via `.Call`. This was repeated 10 times with new matrices, and the average time and standard error were computed for each access method. Row and column access times were evaluated with respect to the numbers of rows and columns, respectively. For sparse matrices, times were also recorded with respect to the density of non-zero entries. In all cases, standard errors were negligible and not plotted. We also ran `gc` prior to each call, to reduce the risk of garbage collection during the timing period.

It is worth noting that all of these timings were performed on a single computer (see Section 3 of S4 Text for details). In practice, the magnitude of differences in access speed between representations depends on many factors such as the amount of microprocessor cache memory, speed of reading data from files on different storage media, etc. These factors are not considered here for simplicity.

2 Performance with ordinary matrices

By default, R stores matrices as one-dimensional arrays of length mn, where m and n are the number of rows and columns, respectively. This is done in column-major format, i.e., the matrix entry (i, j) corresponds to array element $i + mj$ (assuming zero-based indexing). We refer to this format as an ordinary R matrix. The ordinary matrix is easy to manipulate and provides rapid access to row/column data. However, this comes at the cost of random access memory (RAM) usage that is directly proportional to the length of the one-dimensional array. For example, a double-precision matrix containing scRNA-seq data for 10000 genes in each of one million cells would require 80 GB of RAM. This is currently not possible for most workstations, instead requiring dedicated high-performance computing resources. Even smaller matrices will cause problems on systems with limited memory due to R's copy-on-write semantics. Thus, the use of ordinary matrices is limited to relatively small data sets.

We compared the access speed of the *beachmat* API on ordinary matrices to that of a reference implementation using only *Rcpp*. Both row and column access via *beachmat* require 20-50% more time compared to *Rcpp* (S1 Fig). This is because, for each requested row/column, *beachmat* copies the corresponding matrix data into a `Vector` for access by the calling function. The reference implementation avoids the overhead of creating a new copy by simply iterating across the original data. Our use of copying is deliberate as it ensures that the API is consistent across matrix representations – for example, file-based representations *must* copy the data to a new location in RAM. Copying is also required for operations that involve transformations and/or re-ordering of data, as well as for external libraries such as LAPACK that accept a pointer to a contiguous block of memory.

Nonetheless, for read-only access to column data, developers can direct *beachmat* to return an iterator directly to the start of the column of an ordinary matrix. This `get_const_col()` method avoids copying and yields performance equivalent to *Rcpp* (S1 Fig, panel a). For other matrix representations, the same method will simply use the default copy-on-access function `get_col()`. This means that read-only column access can be performed with `get_const_col()` regardless of the representation, while taking advantage of the structure of ordinary matrices for greater efficiency.

3 Performance with sparse matrices

3.1 Overview of the CSC format

The `dgCMatrix` class from the *Matrix* package stores sparse matrix data in compressed sparse column-orientated (CSC) format (S2 Fig). Consider that every non-zero entry in a sparse matrix can be characterized by a triplet of values: row i, column j and data value x. To convert this into the CSC format, entries are sorted in order of increasing $i + mj$. All entries with the same value of j are now grouped together in the ordered sequence. We refer to each column-based group as G_j, the entries of which are sorted internally in order of increasing i. The representation is further compressed by discarding j from each triplet. All entries from the same column lie at consecutive locations of the ordered sequence, so only the start position of G_j on the sequence needs to be stored for each column. (The end position of one column is simply the start position of the next column.) This reduces RAM usage to $s_I n + (s_I + s_D)N_{\neq 0}$ where s_I is the size of an integer, s_D is the size of a single data element and $N_{\neq 0}$ is the number of non-zero elements in the matrix. For double-precision matrices with many rows, sparse matrices will be more memory-efficient than their ordinary counterparts if the density of non-zero elements is less than $\sim 66\%$ (assuming 4-byte integers and 8-byte doubles).

3.2 Timings for column access

The CSC format simplifies data access from sparse matrices by imposing structure on the non-zero entries. When accessing a particular column j, all corresponding entries in G_j can be quickly extracted by taking the relevant stretch of the ordered sequence. For sparse matrices with a low density of non-zero values, column access via *beachmat* is even faster than access from an equivalent ordinary matrix (S3 Fig). This is because only a few non-zero x values need to be copied from the internal data structure – the rest of the output `Vector` can be rapidly filled with zeroes. As the density increases, column access becomes slower but is still comparable to that of ordinary matrices.

As explained above for ordinary matrices, *beachmat* defaults to copy-on-access to retrieve column data from sparse matrices. This is the case regardless of whether `get_col()` or `get_const_col()` is used, as both must explicitly return the zero entries for representation-agnostic behaviour. To improve performance, the *beachmat* API also provides the `get_const_col_indexed()` method to avoid copying from CSC matrices. This returns a tuple containing the number of matrix elements stored in the requested column, an iterator to an integer vector containing the row indices of these elements, and an iterator to a numeric vector containing the values. For CSC matrices, iterators can be directly returned to the internal i and x vectors. For other representations, this method calls `get_const_col()`, which also avoids copying for ordinary matrices while providing representation-agnostic behaviour.

Without copying, *beachmat* is over an order of magnitude faster than the default method for column-level access (S3 Fig). It is also slightly faster than column access through the *RcppArmadillo* and *RcppEigen* APIs [1, 2], which can handle sparse matrices through the `sp_mat` and `MappedSparseMatrix` classes, respectively. We note that part of *beachmat*'s speed-up is caused by the fact that the zero entries of the sparse matrix are completely ignored. Whether or not this is desirable depends on the algorithm being implemented. Indeed, it is possible to use one algorithm for sparse matrices and another for dense matrices, though we only recommend doing so for performance-critical parts of an application. Writing, testing and maintaining two different versions of the same code doubles the burden on the developer, which is precisely what *beachmat* was intended to avoid.

3.3 Timings for row access

Row-level access is more difficult in the CSC format as entries in the same row do not follow a predictable pattern. If a row is requested, a binary search needs to be performed on the values of i in G_j for each column j, which requires an average time proportional to $\log(m)$. In contrast, obtaining the next element in a row of an ordinary matrix can be done in constant time by jumping m elements ahead on the one-dimensional array. To speed up row access for sparse matrices, we realized that the most common access pattern involves requests for consecutive rows. If row i^* is accessed, *beachmat* will loop over all columns and cache the index of the first value of i in G_j that is not less than i^*. When i^*+1 is requested, we simply need to check if each index should be incremented by one. Caching avoids the need for a new binary search and reduces the row access time substantially (Fig 2).

For non-consecutive row access, we mitigate the cost of the binary search by checking if the requested row is greater than or less than the previous row for which indices are stored. If greater, we use the stored indices to set the start of the binary search for the requested row; if less, we use the indices to set the end of the search. This reduces the search space and the amount of computational work for large m, yielding an approximate 2-fold increase in speed when the non-consecutive rows are still ordered (S4 Fig, panel a). Our caching scheme also performs no worse than a naive binary search for random row access (S4 Fig, panel b), and in fact is slightly faster for large m.

The caching procedure increases memory usage slightly as the indices must be stored between row accesses. Specifically, one additional integer must be stored per column, equivalent to the storage of the start position of each G_j group in the CSC format. Generally, the memory overhead of caching is negligible relative to the data stored in the matrix itself, provided that the density of non-zero entries and the number of rows is not very low relative to the number of columns. Nonetheless, *beachmat* will minimize memory usage by not allocating memory for the cache until a row access is requested. This avoids unnecessary memory usage when only column accesses are requested from the API.

Despite these optimisations, row access with sparse matrices in *beachmat* remains slower than that with ordinary matrices (Fig 2a). This is not surprising as there is less work to do when retrieving data from a simpler representation. The exception is with large matrices of low density (Fig 2b), probably because the cost of retrieving non-contiguous blocks of data from RAM for an ordinary matrix exceeds the cost of examining both i and x per non-zero entry in a sparse matrix. In addition, consecutive row access from sparse matrices with *beachmat* is 30-50 times faster than using *RcppArmadillo*. For a 10000-by-1000 sparse matrix with 1% non-zero entries, *beachmat* takes 39.8 milliseconds to access each row and compute the row sums, while *RcppArmadillo* takes 1921.4 milliseconds. These results motivate the use of *beachmat* for general data access from sparse matrices in C++ code.

4 Performance with HDF5-based matrices

For large, non-sparse matrices that do not fit into RAM, the most obvious option is to store the data in a file and load submatrices into memory as required. We consider the use of the hierarchical data format (HDF5) [3], which provides flexible and efficient storage of and access to large amounts of data in a filesystem-like format. Each large matrix is stored as a data set in a HDF5 file, while in R, it

is represented by a `HDF5Matrix` object from the *HDF5Array* package. This object has a very small memory footprint – fewer than 3 kilobytes in size – and simply extracts data from the HDF5 file upon request. Each `HDF5Matrix` instance provides methods to mimic an ordinary matrix, allowing users to manipulate the matrix in real time without needing to load all of the data into memory. Compression of data in the HDF5 file also ensures that the disk space requirements are manageable, even if multiple large matrices need to be generated to hold intermediate results throughout an analysis.

The *beachmat* API supports row- and column-level access from a `HDF5Matrix` instance. Specifically, *beachmat* directly accesses data from the underlying HDF5 file through the official HDF5 C++ API, which has been wrapped in the *Rhdf5lib* package for portability and re-usability in the R package framework. This means that very large data sets can be accessed in C++ with the same code that is used for ordinary and sparse matrix representations. However, data access is inevitably slower than that from an ordinary matrix as the data need to be read from file at regular intervals. We observed a 20-fold increase in the time required for column access and a 40-fold increase for row access (S5 Fig). This suggests that the `HDF5Matrix` representation should be used sparingly – if possible, smaller data sets should use alternative in-memory representations for faster access.

A key determinant of the performance of the `HDF5Matrix` is the layout of data in the HDF5 file. There are two layout choices for large matrices: contiguous or chunked. In the contiguous layout, raw data are flattened into a one-dimensional array analogous to column-major storage of ordinary matrices in memory. (Technically, HDF5 uses a row-major format, but a transposition is internally performed when constructing a `HDF5Matrix`.) In the chunked layout, data are arranged into "chunks" of a pre-defined size. For example, in a row-chunked layout, each chunk would correspond to a row of the matrix. Each chunk is always read from (or written to) file in its entirety by the HDF5 library, even when only a portion of the chunk is requested. Chunking is required for fast access to data, *provided that the layout is consistent with the expected access pattern.* For example, a row-chunked layout allows fast access to each row, as only one chunk needs to be read from file to load the relevant data into memory. However, access to any given column is very slow, as the value of each element in the column must be obtained by reading every row chunk in its entirety, i.e., m reads in total. In practice, both row and column accesses are often required (e.g., to access gene- and cell-level scRNA-seq data), which means that the file layout must be carefully chosen to allow for these orthogonal access patterns.

The choice of file layout is the responsibility of the process that constructs the HDF5 file. This can be the original data provider; the developer whose function returns a `HDF5Matrix`; or a user who coerces their data into a `HDF5Matrix`. As such, the chunking scheme is generally outside of *beachmat*'s control, preventing the API from automatically choosing the optimal layout for the requested access pattern. Nonetheless, for a given layout, *beachmat* will dynamically resize the HDF5 chunk cache to speed up access to consecutive rows or columns (see S2 Text). *beachmat* also provides a function to quickly convert an existing HDF5 file to a layout using pure row- or column-based chunks (see S3 Text, S6 Fig, S7 Fig), which performs well for random row and column access, respectively.

Another benefit of chunking is that the data in each chunk can be compressed using algorithms such as ZLIB and SZIP. In our simulations, the default compression scheme in the *rhdf5* package reduced the size of the HDF5 file by 4-fold for low-precision dense matrices (80 MB to 19 MB for a 10000-by-1000 matrix containing real numbers with two significant figures), with even greater gains for sparse matrices (9 MB for the same matrix with 1% non-zero entries). The use of smaller files reduces the risk that disk space will be exceeded during the course of an analysis. This is important when many `HDF5Matrix` objects need to be constructed, e.g., to store transformed expression values.

5 Other matrix types

While the matrix representations described above are the most commonly used for storing scRNA-seq data, *beachmat* can be easily extended to support other representations. For example, the packed symmetric representation from the *Matrix* package only stores the upper or lower half of a symmetric matrix. This provides an efficient representation of distance matrices, which are often used to cluster

cells based on their expression profiles. *beachmat* supports row and column access of data from packed symmetric matrices through the same interface that is used for the other representations.

beachmat also supports data access from `RleMatrix` instances from the *DelayedArray* package. The `RleMatrix` stores its values as a column-major run-length encoding, where stretches of the same value in the one-dimensional array are stored as a single run. This reduces memory usage in a more general manner than a sparse matrix, especially for matrices with many small but non-zero counts. As with CSC matrices, *beachmat* caches the row indices to speed up consecutive row access.

Another option for constructing file-backed matrices is to use the *bigmemory* package [4]. This constructs an in-memory `big.matrix` object that contains external pointers to a file in which the data are stored. In *beachmat*, we have deliberately chosen `HDF5Matrix` rather than `big.matrix` due to the standardized nature of the HDF5 specification and portability of HDF5 files across systems. Nonetheless, we note that it is simple to extend *beachmat* to accept `big.matrix` inputs if required.

6 Using each representation in matrix multiplication

To explore the performance of *beachmat* for more complex operations, we implemented a matrix multiplication operation in C++. We considered the multiplication of two square matrices of order ranging from 500-2000, using ordinary matrices containing random values from a normal distribution; sparse matrices generated using `rsparsematrix` with 1% density; or HDF5-backed matrices filled with random normal values. Multiplication of low-density sparse matrices was fastest while multiplication of HDF5-backed matrices was slowest (S8 Fig), consistent with our previous access timings. For comparison, timings were also obtained for the `%*%` operator in R (from the base package, for ordinary matrices; from the *Matrix* package, for sparse matrices; or from the *DelayedArray* package, for HDF5-backed matrices). We observed that the performance of our implementation for ordinary and HDF5-backed matrices was comparable to the representation-specific `%*%` operators, despite our use of a naive algorithm that was not cache-optimized. For sparse matrices, a different algorithm that exploits sparsity in both left- and right-side matrices was even faster than `%*%` from *Matrix*.

References

[1] Dirk Eddelbuettel and Conrad Sanderson. RcppArmadillo: Accelerating R with high-performance C++ linear algebra. *Computational Statistics and Data Analysis*, 71:1054–1063, March 2014.

[2] Douglas Bates and Dirk Eddelbuettel. Fast and elegant numerical linear algebra using the RcppEigen package. *Journal of Statistical Software*, 52(5):1–24, 2013.

[3] The HDF Group. Hierarchical Data Format, version 5, 1997-2017. http://www.hdfgroup.org/HDF5/.

[4] Michael J. Kane, John Emerson, and Stephen Weston. Scalable strategies for computing with massive data. *Journal of Statistical Software*, 55(14):1–19, 2013.
