# Supplementary material for: beachmat: A Bioconductor C++ API for accessing high-throughput biological data from a variety of R matrix types
Source: PLoS Comput Biol. 2018 May 3;14(5):e1006135. doi: 10.1371/journal.pcbi.1006135 (PMC5953501; doi:10.1371/journal.pcbi.1006135)
Supplement: S2 Text — (PDF) [file pcbi.1006135.s002.pdf]

S2 Text: optimizing HDF5 chunk cache parameters

Aaron T. L. Lun, Hervé Pagès, Mike L. Smith

April 14, 2018

1 Overview

Matrix data in HDF5 files can be partitioned into chunks [1], where each chunk is a submatrix of the same dimensions. When a row or column of the matrix is requested, the HDF5 library will read all chunks that are overlapped by the requested row/column. Each chunk is read from file in its entirety, even if only a subset of the chunk is overlapped by the requested row/column. This is inefficient as more data needs to be processed than is required for the current request. To improve performance, the HDF5 library will cache a small number of chunks in memory once they are read from file. This means that the cached chunks do not have to be re-read if a neighbouring row/column (overlapping the same set of chunks) is requested. Chunks are then evicted from the cache once the cache size limit is exceeded or if the hash indices of different chunks overlap (see Section 3 below). This behaviour allows efficient access to consecutive rows or columns in the HDF5 file. However, some tuning of the parameters of the HDF5 chunk cache is required to achieve this, which we discuss below.

2 Selecting the size of the chunk cache

Assume that we have a chunk cache containing s data values (this needs to be multiplied by the size of each value in bytes to obtain the actual cache size). Denote the number of rows and columns of our matrix as m and n, respectively. We further assume that these data are stored in a chunked layout, where the number of rows and columns in each chunk is p and q, respectively.

The chunk size s should be chosen so that it can hold all chunks lying across a row or column. Each chunk contains pq values, and each row and column overlaps $\lceil n/q \rceil$ and $\lceil m/p \rceil$ chunks, respectively. This reflects the fact that each chunk is stored in its entirety, even at the right/bottom edges of the matrix where a chunk may not be completely filled. For row access, this means that

$$s \geq \lceil n/q \rceil \, pq \; , \tag{1}$$

i.e., all chunks overlapping a particular row are held in memory. Simliarly, for column access,

$$s \geq \lceil m/p \rceil \, pq \; . \tag{2}$$

This ensures that all chunks overlapping a particular row or column are retained in memory. Otherwise, if s is too small, existing chunks in the cache would be evicted before the current row or column is fully read. This would preclude the use of the cache to rapidly load data for the next row or column.

3 Choosing the number of slots

The chunk cache works by hashing the chunks according to their location in a one-dimensional array. Consider a "meta-matrix" of chunks, where each chunk in the original matrix constitutes one entry in the meta-matrix. A meta-row consists of all chunks along one row of this meta-matrix. Similarly,

a meta-column consists of all chunks along one column of the meta-matrix. Denote the number of meta-rows and meta-columns as m_0 and n_0, respectively. Now, flatten the meta-matrix into a one-dimensional array of chunks, organized in row-major format. The first chunk is assigned a hash index of 1 (technically 0, but we will use 1-based indexing for simplicity here). The next chunk is assigned a hash index of 2, and so on. When the hash index exceeds the number of cache slots v, it is reset to 1 for the next chunk. This continues until a hash index is assigned to all entries of the meta-matrix.

The hash indices are important as two chunks with the same hash index cannot co-exist in the chunk cache. This means that v must be carefully chosen to ensure that all chunks in a meta-row or a meta-column have unique hash indices. Otherwise, an existing chunk in the cache will be evicted by a newer chunk with the same hash index. This would require the re-reading of the data in the older chunk when accessing the next row/column, defeating the purpose of the cache. For a meta-row, guaranteeing unique indices is trivial as v only needs to be chosen to be greater than or equal to n_0.

For a meta-column, more care is required. Consider the first chunk lying in the first meta-column with a hash index of 1. The hash index of 1 will only return to the first meta-column when the sequence $\{1, \ldots, v\}$ has been repeated enough times r such that rv is the smallest multiple of n_0. Prior to this, all assigned values of the first meta-column will be unique. This is obvious because non-unique values of the hash index in the first meta-column would imply an existing repeating structure, which is not possible without repeating the hash index of 1. (The same logic can be applied to any other value of the hash index in any other meta-column.) As the number of rows covered by rv is equal to rv/n_0, we simply need to choose v such that $rv/n_0 \geq m_0$. This ensures that the hash index can never repeat itself for a given meta-column before hash indices are assigned to all entries in the meta-matrix. The most direct approach is to set $v = kn_0 + 1$ where $k \in \mathbf{N}^+$ and $kn_0 + 1 \geq m_0$. This guarantees that the lowest common multiple of v and n_0 is their product, and that $rv/n_0 \geq m_0$.

4 Choosing the chunk dimensions

The preceding text assumes that the chunk dimensions p and q were set beforehand. This is the case for existing HDF5 files, where the chunk layout must be specified during data set creation. However, for new files, we have an opportunity to choose the chunk dimensions to improve data access.

To access data from a particular row, we need to read all chunks overlapped by that row, i.e., $\lceil n/q \rceil$ chunks. Once these are loaded into the cache, no more chunks need to be read from file to access the next $p - 1$ rows, i.e., data are held in memory for all p rows simultaneously. For comparison, consider a layout containing row chunks, i.e., each chunk is an entire row. This provides efficient access to any single row as one chunk obtains all and only that row's data. To access p rows from a row-chunked layout, p chunks would need to be read from file. Thus, for accessing consecutive rows with $p \times q$ chunks, we can read the same number of chunks (or fewer) as the row-chunked layout if

$$\lceil n/q \rceil \leq p \; . \tag{3}$$

The same logic applies to column access, where $\lceil m/p \rceil$ reads are required to store data for q consecutive columns. A layout with pure column chunks would require q reads to access the same data. If

$$\lceil m/p \rceil \leq q \; , \tag{4}$$

we can access data in consecutive columns with the same number of chunk reads (or fewer) as the column-chunked layout. The number of chunks that need to be read is a major factor in HDF5 access speed. By choosing p and q to satisfy Inequalities 3 and 4, we can obtain a file layout that provides fast access for both consecutive rows and columns, comparable to pure row- or column-based chunking.

Obviously, any arbitrarily large p and q will satisfy Inequalities 3 and 4. To avoid this, we use Inequalities 1 and 2 to determine the minimum chunk cache size. The aim is to satisfy all of the inequalities while minimizing s to reduce the memory overhead. In general, this requires some numerical analysis to account for the discrete nature of the dimensions. We obtain some approximate values by treating all variables as continuous, which allows us to satisfy all inequalities when $s \geq \max\{m\sqrt{n}, n\sqrt{m}\}$. Once the smallest s is obtained, the largest p and q are easily determined.

5 Worked example of choosing chunk settings

Assume that we have a matrix with 20000 rows and 50000 columns. Our approach suggests that we should choose s as being greater than $50000\sqrt{20000} \approx 7071068$ for optimal access. We set $s = 8000000$ to simplify the later divisions, which corresponds to an in-memory size of 64 MB for double-precision values. Our chunk dimensions are $p = s/50000 = 160$ and $q = s/20000 = 400$. In practice, one may wish to cap the cache size at the cost of some performance, lest too much memory be requested.

beachmat provides functions to perform the above calculations given m and n. However, *beachmat* will not automatically use the computed p and q as the chunk dimensions for output `HDF5Matrix`. The optimal chunk dimensions depend on the anticipated access patterns, which are unknown to the *beachmat* API at the time of file creation. The choice of p and q above is only well-suited for consecutive row and column accesses, while the actual access pattern might be random (as discussed in S3 Text). Thus, the chunk dimensions must be explicitly chosen by the user/developer. Obviously, this is irrelevant when accessing data from existing HDF5 files where the layout is already fixed.

6 Testing different layouts with simulated data

Here, we demonstrate the effect of the HDF5 file layout on data access speed. For consecutive column access, we considered the performance of *beachmat* with a contiguous layout, column-wise chunks with compression, and row-wise chunks. The contiguous layout is the fastest as all relevant data is obtained in one read from file per column (Fig 3a). Column-wise chunking also involves a single read from file to obtain the chunk corresponding to the requested column, but is likely slower due to the overhead of decompression and chunk caching. Rectangular chunking is slightly slower than column-wise chunks, despite the optimized size of the cache – this is attributable to the time spent collecting data across multiple cached chunks. As expected, column access with row-wise chunks is the slowest.

Timings are reversed for consecutive row access, where row-wise chunking provides the best performance (Fig 3b). This is expected as only one chunk read is required to obtain all data in a single row. Access is much slower with column-wise chunking or a contiguous layout. Importantly, rectangular chunking is only slightly slower than row-wise chunking when the chunk cache is optimized. This highlights the utility of rectangular chunks for both row and column accesses with *beachmat*, compared to the other schemes where performance deteriorates rapidly for non-ideal access patterns.

These timings have a caveat in that the poor performance of row-wise chunks for column access (and vice versa) is not directly caused by multiple redundant reads from file. The data sets used in this simulation are small enough that *beachmat* automatically loads all row-wise chunks (i.e., the entire matrix) into the HDF5 chunk cache when any column is requested, and similarly for all column-wise chunks when any row is requested. Reduced speed is probably due to the need to collect data across many cached chunks, rather than multiple reads. Obviously, caching the entire matrix is not possible for the very large data sets that motivate the use of file-backed representations. For these, further degradation in performance can be expected when the API is forced to re-read chunks from file.

References

[1] The HDF Group. Improving HDF5 compression performance. Technical report, The HDF Group, 2015. https://support.hdfgroup.org/HDF5/doc/TechNotes/TechNote-HDF5-ImprovingIOPerformanceCompressedDatasets.pdf.
