# Supplementary material for: beachmat: A Bioconductor C++ API for accessing high-throughput biological data from a variety of R matrix types
Source: PLoS Comput Biol. 2018 May 3;14(5):e1006135. doi: 10.1371/journal.pcbi.1006135 (PMC5953501; doi:10.1371/journal.pcbi.1006135)
Supplement: S3 Text — (PDF) [file pcbi.1006135.s003.pdf]

S3 Text: dealing with random access from a `HDF5Matrix`

Aaron T. L. Lun, Hervé Pagès, Mike L. Smith

April 19, 2018

The chunk cache is not effective for a random access pattern, as there is no guarantee that the cached data will contain the next requested row or column. (We omit the situation where the entire matrix is loaded into the cache, as this defeats the purpose of file-backed storage.) In such cases, pure row- or column-based chunks may be necessary for optimal performance (S6 Fig). However, the best layout is not clear at the time of file creation, especially for scRNA-seq data where it is sensible to access data by gene (row) or cell (column). Switching from one layout to the other may be necessary throughout the course of an analysis that performs random queries on both rows and columns.

The *beachmat* strategy implements a simple approach to converting an existing HDF5 file to a layout using purely row- or column-based chunks. Consider an existing file with chunk dimensions of (p_1, q_1). Assume that we want to obtain a new file with chunk dimensions of $(1, q_2)$, i.e., row-wise chunks containing q_2 values. We extract a submatrix of size (p_1, q_2) from the existing file and write it to the new file with the row-chunked layout. This is repeated with the next submatrix of the same size along the next q_2 columns, and the process is iterated until all columns have been read and written for these p_1 rows. (In cases where q_2 is not a multiple of q_1, we configure the HDF5 chunk cache to hold the input chunk that has not been completely read between iterations.) We repeat this procedure with the next p_1 rows until the entire file has been processed. Conversely, to convert to column-based chunks of size $(p_2, 1)$, a submatrix of size (p_2, q_1) is extracted and written to file; this is repeated for the next p_2 rows, until all rows have been processed for these q_1 columns; and the process is repeated with the next q_1 columns until the entire file is rewritten. The idea is to read and write entire chunks as much as possible, reducing the number of redundant reads from file. Only a submatrix of the data needs to be held in memory at each iteration, which reduces the overhead of the conversion.

Converting from one layout to the other with the above strategy is fast, requiring only a few seconds for small matrices (S7 Fig). The computational time scales linearly with matrix size, so for a large scRNA-seq data set with a million cells, we estimate that the conversion would take 2-3 hours. This is minor compared to the time spent by the entire analysis. Nonetheless, to maximize efficiency, all functions requiring column-based access (e.g., cell-based quality control and normalization) should be grouped together and executed first; this should be followed by a layout conversion step, and then by execution of functions requiring row-based access, e.g., calculation of gene-wise statistics.

An alternative strategy is to store a matrix in both row- and column-chunked form as two separate HDF5 data sets in the same file. Ideally, both layouts would be referenced by a single R object, allowing the user to perform efficient random access to rows and columns simultaneously. It would be similarly straightfoward for *beachmat* to choose the most appropriate layout for data access, depending on whether a row or column is requested. Obviously, though, this approach involves writing and storing an additional data set, doubling the size of the HDF5 file and slowing the creation of the matrix representation. The simultaneous use of two layouts seems to be most suitable for analyses involving a read-only matrix that is written to file beforehand, subsequently providing rapid access to its data while avoiding issues with slower creation of larger files throughout the course of the analysis.
