# Supplementary material for: beachmat: A Bioconductor C++ API for accessing high-throughput biological data from a variety of R matrix types
Source: PLoS Comput Biol. 2018 May 3;14(5):e1006135. doi: 10.1371/journal.pcbi.1006135 (PMC5953501; doi:10.1371/journal.pcbi.1006135)
Supplement: S4 Text — (PDF) [file pcbi.1006135.s004.pdf]

S4 Text: methods for real data processing

Aaron T. L. Lun, Hervé Pagès, Mike L. Smith

April 14, 2018

1 Access timings with the Zeisel *et al.* brain data set

Mouse brain scRNA-seq data [1] were obtained as a count matrix from `http://linnarssonlab.org/cortex`. Counts were read into R and converted into a double-precision simple matrix, a `dgCMatrix` or a `HDF5Matrix`. For each representation, timings of the calculation of row or column sums were performed as previously described. This was repeated 10 times to obtain an average time. Column- and row-wise chunking were used for timing column and row access, respectively, of a `HDF5Matrix`.

We also timed the calculation of other metrics such as the number of detected genes per cell and the number of expressing cells per gene. This was achieved by writing custom C++ functions based on *beachmat* using no-copy column access methods, i.e., `get_const_col_indexed()`. For each metric, we recorded the time required as previously described, and compared to implementations written in R using `rowSums` or `colSums` after coercion to a logical matrix. We used the base `rowSums`/`colSums` methods for simple matrices, methods in *Matrix* for sparse matrices, and methods in *DelayedMatrixStats* (`https://bioconductor.org/packages/DelayedMatrixStats`) for HDF5-backed matrices. We repeated the timings for the library size for each cell with *beachmat* or R's `colSums`. Note that this differs from the timings of the column sums above, which uses the default copy-on-access methods.

2 Analysis of the 1.3 million brain cell data set

We obtained the 1.3 million brain cell data set from the 10X Genomics website (`https://support.10xgenomics.com/single-cell-gene-expression/datasets/1.3.0/1M_neurons`) and converted it into a `HDF5Matrix` object with rectangular chunks using the *TENxGenomics* package (`https://github.com/mtmorgan/TENxGenomics`). We wrapped this object in the *TENxBrainData* package (`https://bioconductor.org/packages/TENxBrainData`) for convenient access during analysis. We used the `calculateQCMetrics` function from *scater* [2] to compute quality control metrics for each cell, including the total number of unique molecular identifiers (UMIs) and the total number of expressed genes (i.e., with at least one UMI). Low-quality cells were identified as those with log-total UMI counts or log-numbers of expressed genes that were more than three median absolute deviations below the median for all cells in the same sequencing library, and were filtered out prior to further analyses.

To assign cell cycle phase to each cell, we applied the `cyclone` method [3] from the *scran* package using the pre-defined mouse classifier. This was performed over three cores to reduce computational time. To compute size factors, we used the the deconvolution method [4]. We performed pre-clustering of cells within each sequencing library, using a shared-nearest-neighbour clustering approach [5] on the rank-based distances. This yielded clusters containing 200-3000 cells each. Deconvolution was applied to each cluster using the `computeSumFactors` function, and size factors were calibrated across clusters by normalizing the cluster-specific pseudo-cells. This procedure avoids violating the non-DE assumption when pooling many different cell types. The size factor for each cell was used to compute normalized log-expression values [6], which were stored in a new `HDF5Matrix` object.

To identify highly variable genes (HVGs), we used the `trendVar` and `decomposeVar` functions from *scran*. We computed the variance of the normalized log-expression values for each gene while blocking

on the sequencing library of origin. We fitted a mean-dependent trend to the variances to model the mean-variance relationship, using a loess smoother with a span of 0.05 and 100 robustness iterations. We defined HVGs as those genes with observed variances above the fitted value of the trend at the same abundance. For comparison, we also constructed the mean-variance trend corresponding to Poisson noise, representing the technical variance of sequencing [7] or capture for the UMI counts.

We performed randomized PCA [8] on the expression matrix for all cells, using the `rsvd` function in the *BigDataAlgorithms* package (`https://github.com/Bioconductor/BigDataAlgorithms`). This provides a `HDF5Matrix`-compatible version of the randomized SVD implementation in the *rsvd* package (`https://cran.r-project.org/package=rsvd`). Specifically, we mean-centered the expression matrix and filtered for HVGs before applying `rsvd` with a rank of 50. Coordinates in the PC space for each cell were computed from the U and D matrices, and the percentage of variance explained was computed from the diagonal elements of D. Note that this function relies on a block processing strategy to matrix multiply `HDF5Matrix` objects – *beachmat* and C++ were not used. We chose to use randomized PCA due to its speed – on a server with 1 TB of RAM, randomized PCA required only 2.2 hours to compute the first 50 dimensions for an ordinary matrix containing 10% of the data set. By comparison, PCA based on the standard SVD failed to complete on this subset after 310 hours.

3 Details of the computing environment

All timings and analyses (except for the comparison between randomized and standard SVD) were performed on a Dell OptiPlex 790 desktop with an Intel Core i5 processor and 8 GB of RAM, running Ubuntu 14.04.5 with R-devel (3.5) and packages from Bioconductor 3.7. On this machine, the 10X data analysis required approximately 4 hours for quality control; 22 hours for cell cycle phase assignment (using three cores); 12 hours for calculation of size factors and generation of normalized expression values; 24 hours for detecting highly variable genes; and 30 hours for the randomized PCA.

References

[1] A. Zeisel, A. B. Munoz-Manchado, S. Codeluppi, P. Lonnerberg, G. La Manno, A. Jureus, S. Marques, H. Munguba, L. He, C. Betsholtz, C. Rolny, G. Castelo-Branco, J. Hjerling-Leffler, and S. Linnarsson. Brain structure. Cell types in the mouse cortex and hippocampus revealed by single-cell RNA-seq. *Science*, 347(6226):1138–1142, Mar 2015.

[2] D. J. McCarthy, K. R. Campbell, A. T. Lun, and Q. F. Wills. Scater: pre-processing, quality control, normalization and visualization of single-cell RNA-seq data in R. *Bioinformatics*, 33(8):1179–1186, Apr 2017.

[3] A. Scialdone, K. N. Natarajan, L. R. Saraiva, V. Proserpio, S. A. Teichmann, O. Stegle, J. C. Marioni, and F. Buettner. Computational assignment of cell-cycle stage from single-cell transcriptome data. *Methods*, 85:54–61, Sep 2015.

[4] A. T. Lun, K. Bach, and J. C. Marioni. Pooling across cells to normalize single-cell RNA sequencing data with many zero counts. *Genome Biol.*, 17:75, Apr 2016.

[5] C. Xu and Z. Su. Identification of cell types from single-cell transcriptomes using a novel clustering method. *Bioinformatics*, 31(12):1974–1980, Jun 2015.

[6] A. T. Lun, D. J. McCarthy, and J. C. Marioni. A step-by-step workflow for low-level analysis of single-cell RNA-seq data with Bioconductor. *F1000Res*, 5:2122, 2016.

[7] J. C. Marioni, C. E. Mason, S. M. Mane, M. Stephens, and Y. Gilad. RNA-seq: an assessment of technical reproducibility and comparison with gene expression arrays. *Genome Res.*, 18(9):1509–1517, Sep 2008.

[8] N. Halko, P. G. Martinsson, and J. A. Tropp. Finding structure with randomness: Probabilistic algorithms for constructing approximate matrix decompositions. *SIAM Review*, 53(2):217–288, 2011.
