# Supplementary figures and images for: beachmat: A Bioconductor C++ API for accessing high-throughput biological data from a variety of R matrix types
Source: PLoS Comput Biol. 2018 May 3;14(5):e1006135. doi: 10.1371/journal.pcbi.1006135 (PMC5953501; doi:10.1371/journal.pcbi.1006135)

Column access

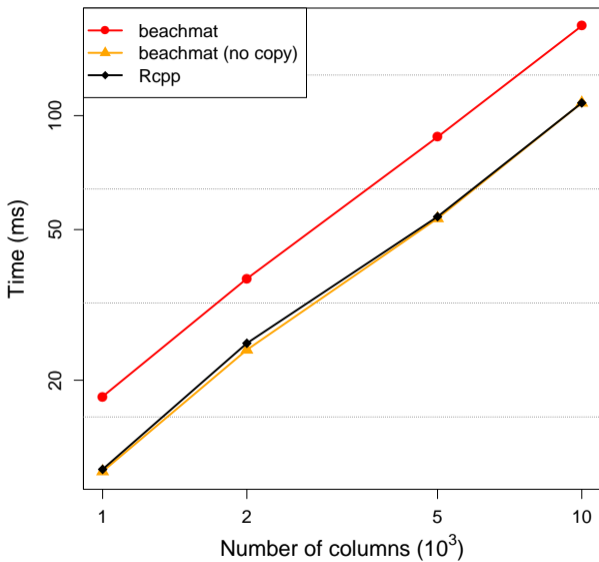

(a)

Row access

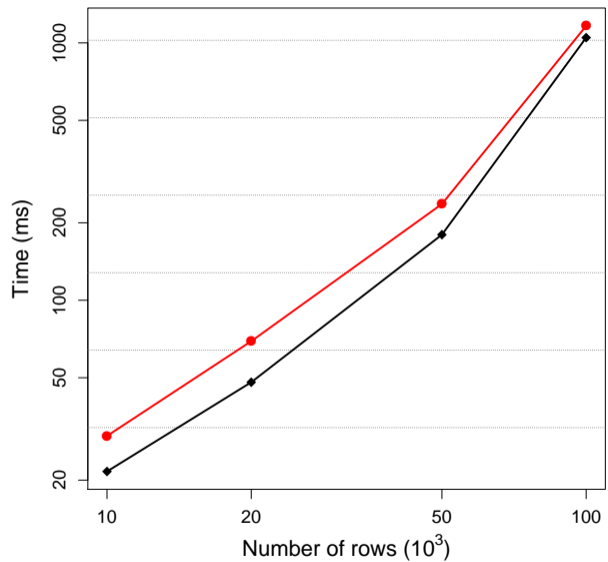

(b)

Supplement: S1 Fig — (a) Column access time with respect to the number of columns, for a matrix with 10000 rows. Times are shown for beachmat with and without copying of matrix data. (b) Row access time with respect to the number of rows, for a matrix with 1000 columns. Each timing represents the average of 10 simulations, and involves accessing the entirety of the matrix. Intervals between the horizontal dotted lines represent 2-fold increases in time. (PDF) [file pcbi.1006135.s005.pdf]

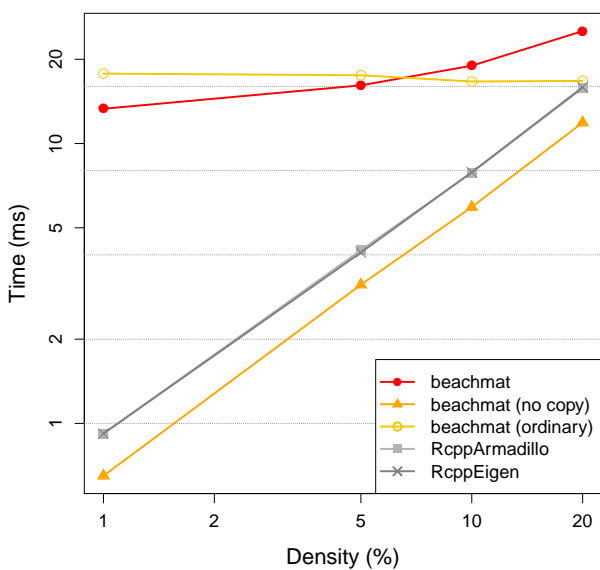

(a)

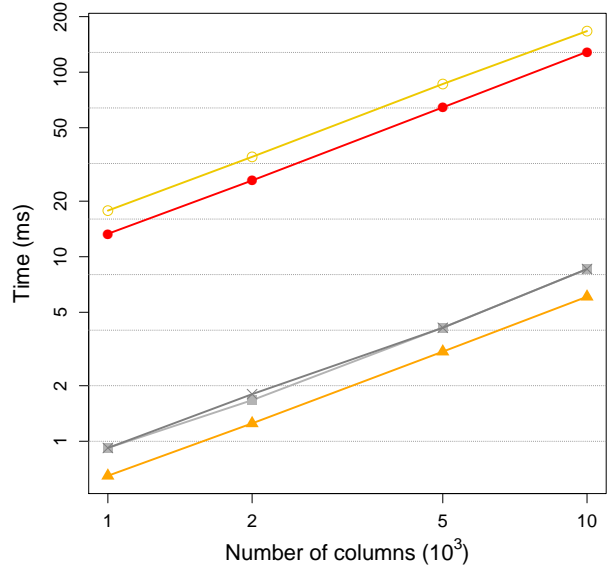

(b)

Supplement: S3 Fig — Timings were also recorded for a copy-free column access method for CSC matrices in beachmat. (a) Access times with respect to the density of non-zero entries as a percentage of all entries, for a matrix with 10000 rows and 1000 columns. (b) Access times with respect to the number of columns, for a matrix with 10000 rows and 1% density. Each timing represents the average of 10 simulations, and involves accessing all columns in the matrix. Horizontal dotted lines represent 2-fold increases in time. (PDF) [file pcbi.1006135.s007.pdf]

Ordered

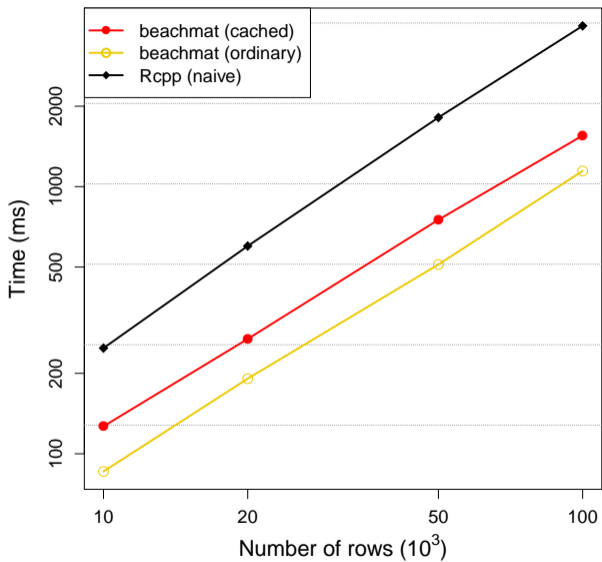

(a)

Random

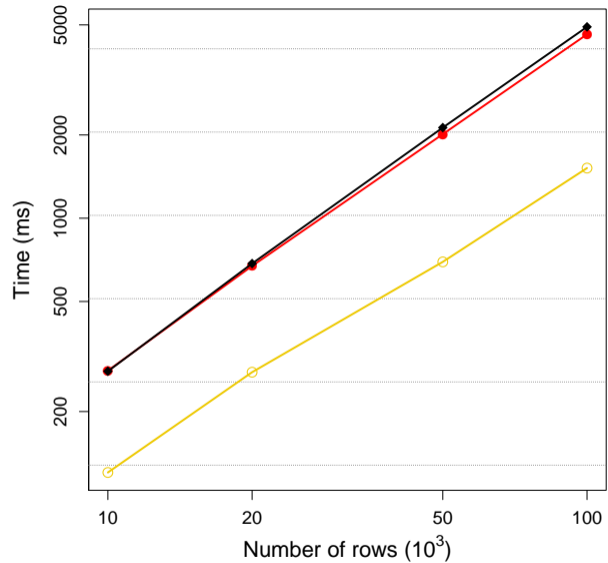

(b)

Supplement: S4 Fig — (a) Access times for ordered but non-consecutive rows, with respect to the number of rows in a matrix with 10000 rows and 1000 columns at 1% density. This involved accessing every 5th row and returning to the first unaccessed row, i.e., {1, 6, …, 9996, 2, 7, …}. (b) Access times for random rows, with respect to the number of rows in the matrix described previously. Each timing represents the average of 10 simulations, and involves accessing all rows in the matrix exactly once. Horizontal dotted lines represent 2-fold increases in time. (PDF) [file pcbi.1006135.s008.pdf]

Column access

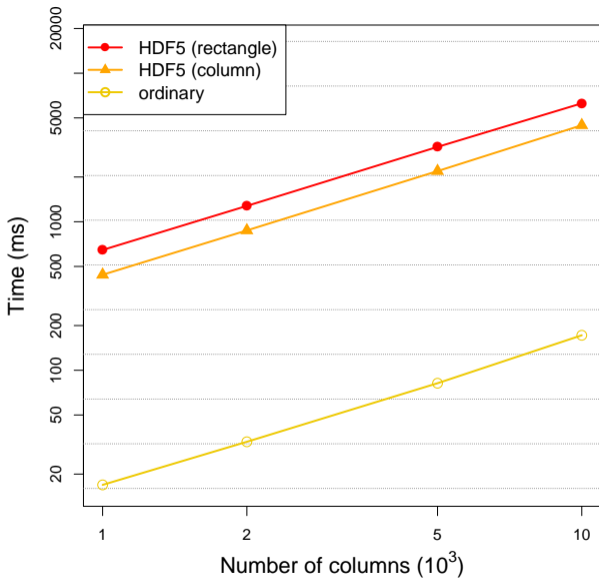

(a)

Row access

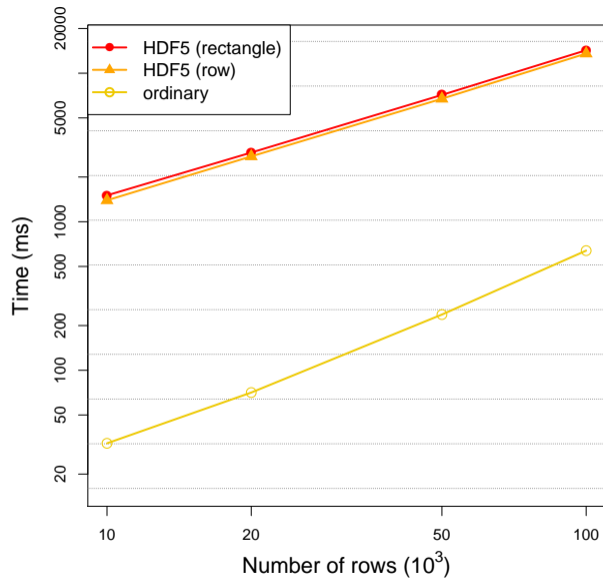

(b)

Supplement: S5 Fig — (a) Column access time with respect to the number of columns, for a dense matrix with 10000 rows. (b) Row access time with respect to the number of rows, for a dense matrix with 1000 columns. Each timing represents the average of 10 simulations, and involves accessing the entirety of the matrix. Horizontal dotted lines represent 2-fold increases in time. (PDF) [file pcbi.1006135.s009.pdf]

Column access

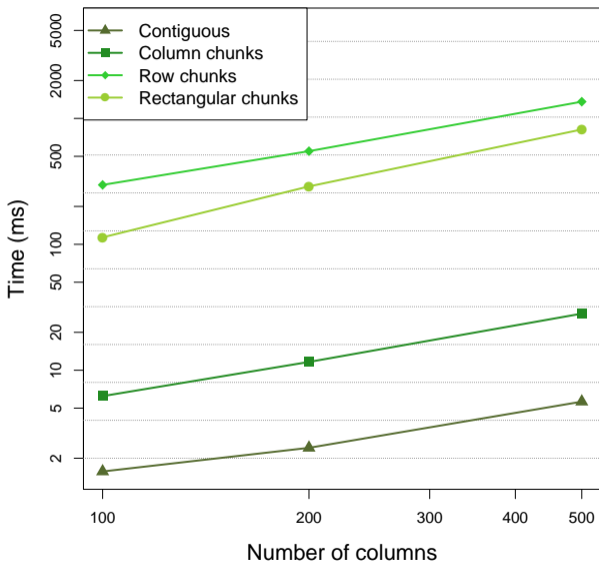

(a)

Row access

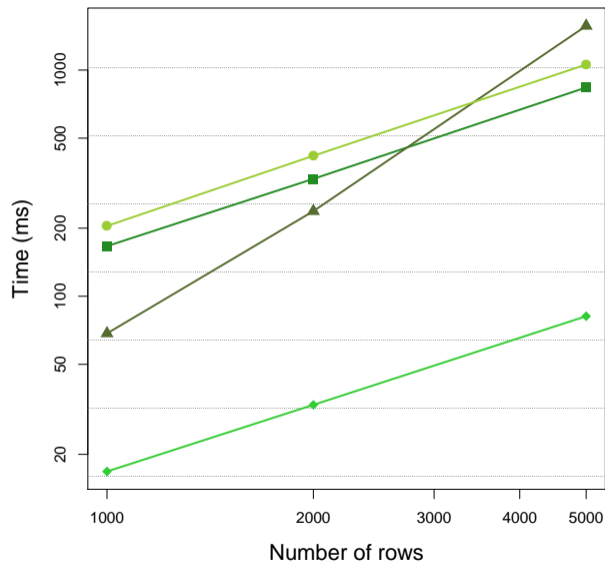

(b)

Supplement: S6 Fig — (a) Random column access times with respect to the number of columns, for a dense matrix with 1000 rows. (b) Random row access times with respect to the number of rows, for a dense matrix with 100 columns. Each row or column in the matrix was accessed exactly once in random order. Horizontal dotted lines represent 2-fold increases in time. (PDF) [file pcbi.1006135.s010.pdf]

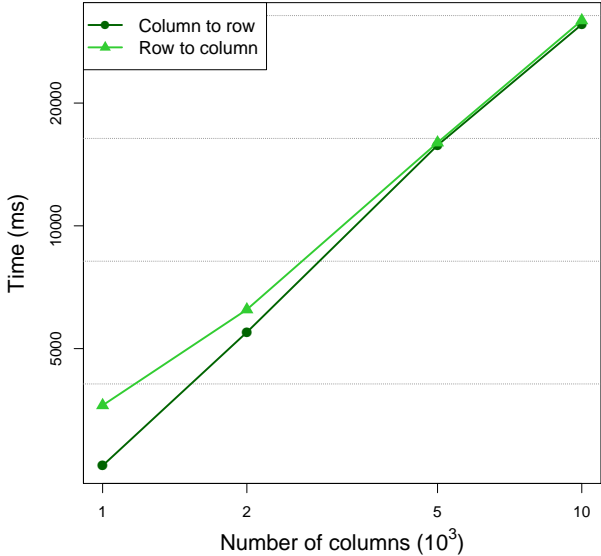

(a)

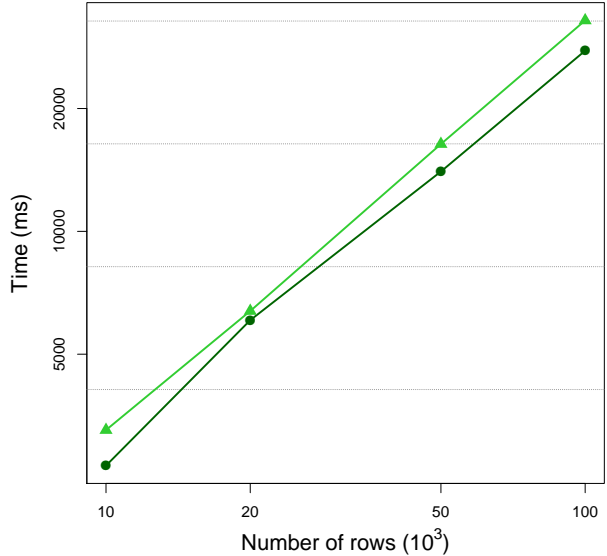

(b)

Supplement: S7 Fig — Each chunk contained 5000 values along a single row or column (or set to the corresponding dimension of the matrix, if it was smaller than 5000). Conversion times were recorded with respect to increasing number of (a) columns for a dense matrix with 10000 rows, or (b) rows for a dense matrix with 1000 columns. All values represent the mean of 10 simulation iterations. Horizontal dotted lines represent 2-fold increases in time. (PDF) [file pcbi.1006135.s011.pdf]

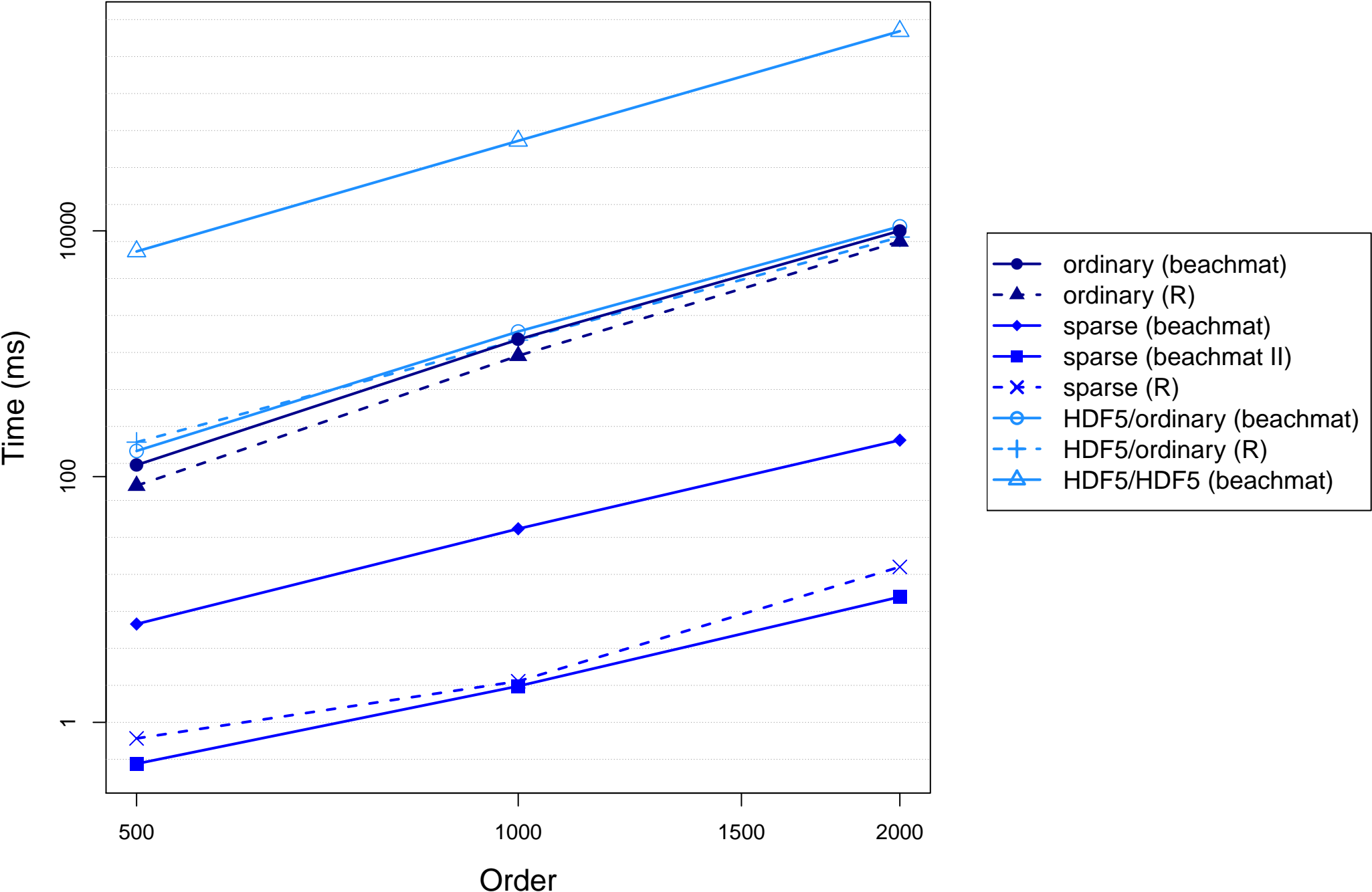

Supplement: S8 Fig — Matrix multiplication was performed using a simple algorithm implemented in C++ with beachmat, or the representation-specific %*% operators in R. For sparse matrix multiplication, timings are also provided for an alternative algorithm implemented in beachmat that better exploits sparsity (II). Timings for the multiplication of two HDF5-backed matrices are shown for beachmat only, as the equivalent operation is not yet supported by DelayedArray. (PDF) [file pcbi.1006135.s012.pdf]
