# Supplementary material for: beachmat: A Bioconductor C++ API for accessing high-throughput biological data from a variety of R matrix types
Source: PLoS Comput Biol. 2018 May 3;14(5):e1006135. doi: 10.1371/journal.pcbi.1006135 (PMC5953501; doi:10.1371/journal.pcbi.1006135)
Supplement: S2 Fig — The grey box represents a sparse matrix with zero entries indicated by the dots. The x vector stores all non-zero values, ordered in column-major format. The index of each element in x is shown in red. The i vector stores the row indices (blue) corresponding to the ordered non-zero values. The p vector stores the element index of the first non-zero value in each column (brown). The last element of p is always the total number of non-zero entries. (PDF) [file pcbi.1006135.s006.pdf]

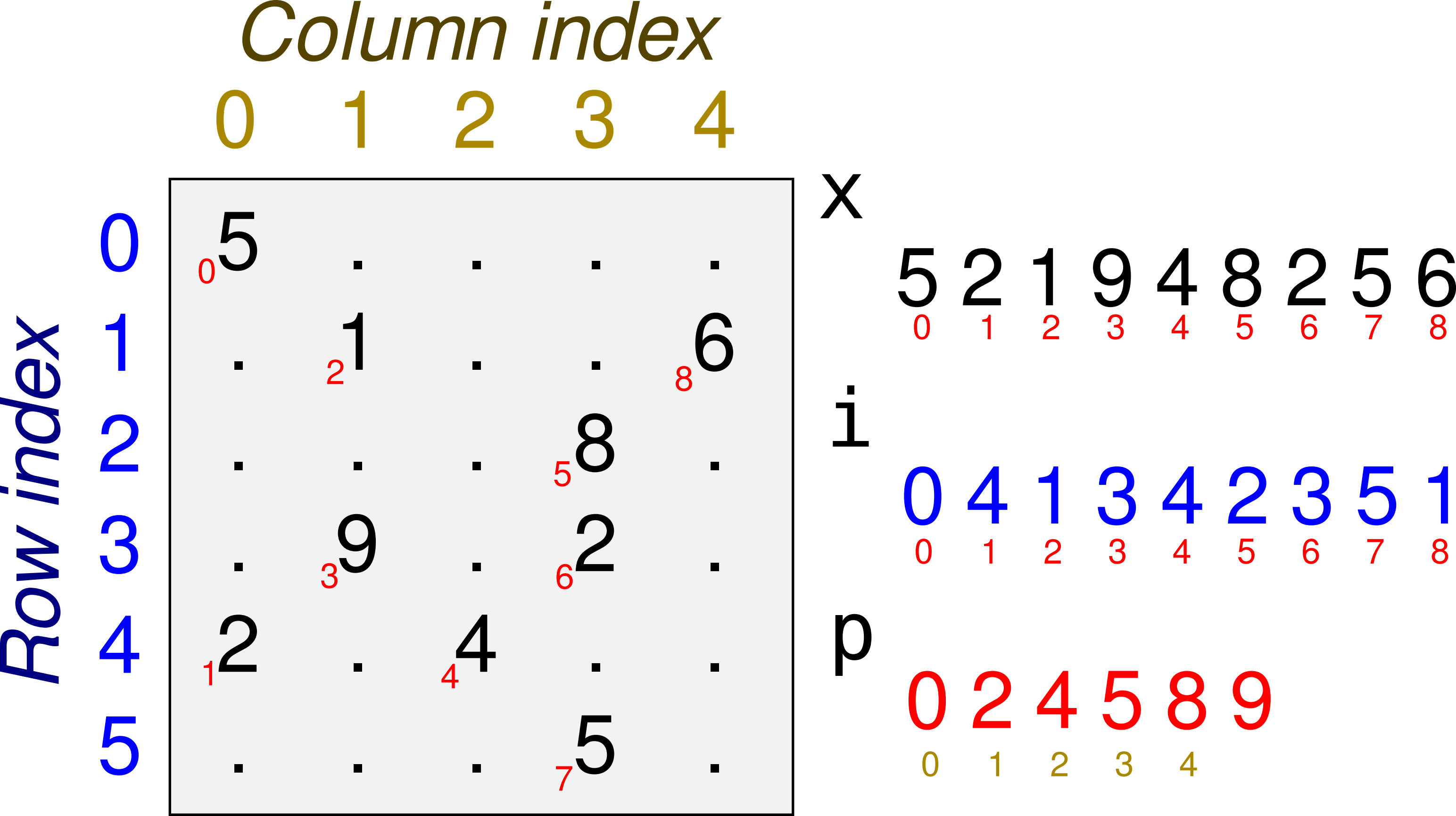
Column index
0 1 2 3 4
Row index
0 1 2 3 4 5
x
5 2 1 9 4 8 2 5 6
0 1 2 3 4 5 6 7 8
i
0 4 1 3 4 2 3 5 1
0 1 2 3 4 5 6 7 8
p
0 2 4 5 8 9
0 1 2 3 4
